# Leveraging Cognitive and Speech Ecological Momentary Assessment in Individuals With Phenylketonuria: Development and Usability Study of Cognitive Fluctuations in a Rare Disease Population

**DOI:** 10.2196/63644

**Published:** 2025-06-03

**Authors:** Shifali Singh, Lisa Kluen, Katelin Curtis, Raquel Norel, Carla Agurto, Elizabeth Grinspoon, Zoe Hawks, Shawn Christ, Susan Waisbren, Guillermo Cecchi, Laura Germine

**Affiliations:** 1 McLean Hospital Harvard Medical School Belmont, MA United States; 2 Digital Health IBM Research Yorktown Heights, NY United States; 3 Department of Psychological Sciences University of Missouri Columbia, MO United States; 4 Genetics and Metabolism Program, Boston Children’s Hospital Department of Pediatrics, Harvard Medical School Boston, MA United States

**Keywords:** neuropsychology, ecological momentary assessment, rare diseases, metabolism, cognition, phenylketonuria, PKU, hereditary, phenylalanine hydroxylase deficiency, phenylalanine

## Abstract

**Background:**

Phenylketonuria (PKU) is a rare, hereditary disease that causes disruption in phenylalanine (Phe) metabolism. Despite early intervention, individuals with PKU may have difficulty in several different cognitive domains, including verbal fluency, processing speed, and executive functioning.

**Objective:**

The overarching goal of this study is to characterize the relationships among cognition, speech, mood, and blood-based biomarkers (Phe, tyrosine) in individuals with early treated PKU. We describe our initial optimization pilot results that are guiding this study while establishing the feasibility and reliability of using ecological momentary assessment (EMA) in this clinical population.

**Methods:**

In total, 20 adults with PKU were enrolled in this study between December 2022 and March 2023 through the National PKU Alliance. Of the total, 18 participants completed an extended baseline assessment followed by 6 EMAs over 1 month. The EMAs included digital cognitive tests measuring processing speed, sustained attention, and executive functioning, as well as speech (semantic fluency) and mood measures. Participants had 60 minutes to complete the assessment.

**Results:**

Completion rates of EMAs were above 70% (on average 4.78 out of 6 EMAs), with stable performances across baseline measures and EMAs. Between-person reliability (BPR) of the EMAs, representing the variance due to differences between individuals versus within individuals, is satisfactory with values close to (semantic fluency BPR: 0.7, sustained attention BPR: 0.72) or exceeding (processing speed: 0.93, executive functioning: 0.88) data collected from a large normative database (n=5039-10,703), as well as slightly below or matching a previous study using a clinical group (n=18). As applicable, within-person reliability was also computed; we demonstrated strong reliability for processing speed (0.87). A control analysis ensured that time of day (ie, morning, afternoon, and evening) did not impact performance; performance on tasks did not decrease if tested earlier versus later in the day (all P values >.09). Similarly, to assess variability in task performance over the course of all EMAs, the coefficient of variability was computed; 28% for the task measuring sustained attention, 37% for semantic fluency, 15.8% for the task measuring executive functioning, and 17.6% for processing speed. Performance appears more stable in tasks measuring processing speed and executive functioning than on tasks of sustained attention and semantic fluency.

**Conclusions:**

Preliminary results of this study demonstrate strong reliability of cognitive EMA, indicating that EMA is a promising tool for evaluating fluctuations in cognitive status in this population. Future work should refine and expand the utility of these digital tools, determine how variable EMA frequencies might better characterize changes in functioning as they relate to blood-based biomarkers, and validate a singular battery that could be rapidly administered at scale and in clinical trials to determine the progression of disease.

## Introduction

### Phenylketonuria and Cognition

Each year, 6 out of 100,000 newborns are diagnosed with phenylketonuria (PKU) [1]. PKU is a rare, hereditary disease [2,3] that, if left untreated, can lead to severe brain damage, intellectual disabilities, and behavioral issues [1,3,4]. PKU is characterized by a deficiency in the phenylalanine hydroxylase enzyme, necessary for the metabolism of the amino acid or phenylalanine (Phe) [3,5-7]. This causes disruptions in Phe metabolism [2] and deficiencies in tyrosine, or Tyr, with significant downstream effects on serotonin and dopamine [5,7]. Individuals with PKU tend to experience difficulties on tests measuring verbal fluency [2,8], processing speed [3,9], and executive functioning [5,6]. In addition, despite early intervention, patients with PKU also typically exhibit lower IQ scores, but to a lesser degree than executive functioning and processing speed [1,3,4,9].

### PKU Biomarkers and Cognitive Functioning

Cognitive functioning in patients with PKU is greatly affected by fluctuations in blood Phe and blood Tyr levels. Although the relationship between Phe and cognitive functioning is more like a continuum, such that higher Phe is associated with greater impairment in cognitive functioning, in general, patients with PKU with Phe levels above 600 mmol/L demonstrate impaired cognition [3,4] and poor frontal lobe function [10]. Participants with even higher Phe levels, above 1000 mmol/L, scored lower on a greater number of cognitive tests assessing cognitive domains; this includes attention, verbal fluency, reaction time, verbal recognition memory, visual memory, and naming, compared with those with Phe levels lower than 1000 mmol/L [11,12]. Greater variability in Phe levels also appears to contribute to the severity of neurocognitive sequelae [3]. Indeed, previous work points to variability in Phe control as the strongest predictor of executive function and general cognitive outcomes, wherein Phe variability may be a better indicator of cognitive functioning than both metabolic control and age [3].

### Assessing Fluctuations in Cognitive Status

Most previous studies of neurocognitive functioning in individuals with PKU have relied on traditional neuropsychological evaluations, which only capture a single time point of assessment, or a “snapshot” of cognitive functioning. This is not necessarily problematic, as this is consistent with standard clinical practice; however, these assessments are often limited in that individuals with PKU may experience fluctuations in cognitive status, wherein their cognition may shift within hours, days, weeks, and months. Thus, a typical evaluation is unlikely to capture this variance adequately [13]. Thus, we use a methodological approach known as ecological momentary assessment (EMA) in this study; this facilitates repeated assessments over time to capture intraindividual and interindividual variability [14-16]. EMA has been particularly effective with the widespread use of smartphones, allowing real-time capture of behavioral, psychological, and cognitive processes. Furthermore, it is a particularly [17] robust method of data capture given that it enables investigators to account for contextual and environmental factors, which are typical limitations of traditional research methodologies that most frequently capture functioning in a lab at a single time point, without any external distractions [18,19].

Previous work has demonstrated that EMA can be used to evaluate everyday fluctuations in *cognitive status*, enabling assessment of cognitive functioning in naturalistic contexts [20,21]. Yet this approach is just recently being incorporated into studies with patients diagnosed with PKU, despite EMA being an effective tool to measure everyday variability, or fluctuations, in cognitive status [3,17,22]. EMA is an ideal methodology for the study of rare disease populations like PKU, because it allows for the collection of multiple data points from each individual by increasing reliability and improves statistical power to detect clinically meaningful findings with small sample sizes—a key factor in rare disease populations. In addition, EMA study designs enable detailed analyses of multiple dynamic biological, cognitive, and psychological processes, such that one can effectively characterize specific clinical populations [23-25].

### This Study

The primary overarching goal of this study is to characterize the relationships among cognition, speech, mood, and blood-based biomarkers (Phe, Tyr) in individuals with early treated PKU. This study, led by principal investigator SS of McLean Hospital of Harvard Medical School and funded by the Phenylalanine Families and Researchers Exploring Evidence (PHEFREE) Consortium, leverages EMA to evaluate real-time cognitive status, speech or voice biomarkers, and psychological functioning. Blood-based biomarkers, including Phe and Tyr, are also useful in contextualizing these relationships and were assessed on the days of EMA administration; further work on this project will aim to gather more Phe and Tyr data to better establish psychometric robustness. Participants are continuing to be recruited for this study.

This paper reports results from an initial EMA optimization pilot of 20 participants diagnosed with PKU. This study was conducted before the completion of a longer protocol to determine the most optimal battery for individuals with PKU, including the number, length, and frequency of EMAs. We describe our initial optimization pilot results that are guiding the study design. We collected EMAs 6 times within a month; finger prick tests were completed on the day of each EMA to determine how variations in amino acid metabolism might relate to or predict fluctuations in certain aspects of functioning. This frequency was chosen to limit participant burden while still accounting for a wide range of data capture, varying between time of day and day of week over 1 month. Our goal is to determine whether the current method of evaluation, using EMA, is appropriate for individuals diagnosed with PKU, as well as how performance on cognitive tests might vary over time. This has implications for widespread dissemination of rapid, repeatable, scalable batteries that can be administered completely remotely, offering greater equity and accessibility in evaluating individuals with PKU on an international scale.

## Methods

### Participants

All participants were recruited through flyers distributed by the National PKU Alliance (NPKUA) through email with individuals in their patient registry and by postings on social media platforms associated with the organization. Recruitment specifically targeted individuals already enrolled in the NPKUA registry, a database that connects individuals diagnosed with PKU because it aligns with the study’s objective of examining the cognitive and behavioral impacts of the rare disease. Recruitment materials provided detailed information about the study and instructions for those who expressed interest in participation. To be eligible, participants had to meet the following inclusion criteria: current US resident, normal or corrected-to-normal vision, ability to provide consent, and a diagnosis of PKU. Participants were excluded based on the following: significant physical disabilities affecting their ability to perform digital assessments (eg, due to visual, motor, or hearing impairments) or their inability to complete EMAs during the study period (eg, due to planned travel, night shift work, or occupation that does not allow time to complete assessments within 60 minutes). Potential participants were screened during an initial virtual visit to confirm eligibility. The study’s purpose, procedures, risks, and expectations were thoroughly explained. Informed consent was collected electronically via a secure REDCap (Research Electronic Data Capture; Vanderbilt University) form before enrollment.

### Materials

#### Baseline Assessment

Baseline cognitive tests, speech assessments, and psychological questionnaires were completed by all participants via their smartphones upon enrollment in the study. We used two platforms, including (1) TestMyBrain (TMB), which is an open-source, digital cognitive test platform with data collected from approximately 3 million participants worldwide [20,22,26-28], and (2) SurveyLex, developed by Sonde Health, which is a speech acquisition platform that enables voice recordings in response to predefined prompts or free response questions. This tool has been used to effectively validate voice biomarkers in other work [29]. Tasks were selected based on traditional neuropsychological evaluations, as well as speech measures typically used by collaborators on this project from the IBM Thomas J. Watson Research Center. In total, the baseline battery took approximately 90 minutes to complete and included “full versions” of all cognitive EMAs (refer to Table 1 for a complete list); of note, this is significantly longer than the ultrabrief, EMA versions of cognitive tests used for repeated assessments throughout the study. Please refer to Table 1 for a complete list of baseline assessments and their descriptions.

**Table 1:** Baseline assessments and constructs measured.

Questionnaire or assessment			Description
**Baseline questionnaires (approximately 20 min)**			
	General questionnaire	Demographic characteristics, sleep and wake times in a typical work week, and employment.	
	PROMIS^a^ Scale [30]	Questionnaire assessing self-reported anxiety and depression using the PROMIS Short Form v1.0 Anxiety 7a and PROMIS Short Form v1.0 Depression 8b scales. Together, they form a combined 15-item measure.	
	Global perceived stress scale [31]	Questionnaire assessing chronic experiences of stress. It is a 10-item scale measuring the degree to which situations are appraised as stressful. It takes approximately 5 min.	
	Quality of Life in Neurological Disorders (Neuro-QoL)—Cognitive Function Short Form [32]	Questionnaire assessing self-reported cognitive problems in daily life. It is an 8-item questionnaire and takes approximately 5 min.	
	Mental Health Questionnaire [33,34]	Questionnaire assessing cross-cutting symptoms for psychopathology based on the *Diagnostic and Statistical Manual of Mental Disorders, Fifth Edition*. It is a 6-item questionnaire assessing possible broad psychopathology and takes approximately 2 min.	
	World Health Organization Alcohol, Smoking and Substance Involvement Screening Test (ASSIST) [35]	Screening for alcohol consumption, smoking, and other substance use over lifetime and the last 3 mo before the assessment. It takes approximately 3 min.	
	Snoring, tiredness, observed apnea, high blood pressure, BMI, age, neck circumference, and male gender (STOP-Bang) Questionnaire [36]	Questionnaire assessing obstructive sleep apnea risk. It is an 8-question measure and takes approximately 2 min.	
**Baseline cognitive assessment (approximately 60 min)**			
	TMB^b^ website simple reaction time	Cognitive test assessing basic psychomotor speed. Participants press a button every time a green square appears on screen.	
	TMB vocabulary	Cognitive test assessing verbal reasoning. Participants indicate which of the 5 words is the closest in meaning to a target word.	
	TMB digit symbol matching (DSM)	Cognitive test assessing psychomotor processing speed. Participants match a set of symbols to the numbers 1, 2, or 3 based on a key presented on screen.	
	TMB gradual onset continuous performance test (gradCPT)	Cognitive test assessing sustained attention. Participants see a series of city or mountain scenes and are asked to press a button whenever they see a city scene and withhold a response whenever they see a mountain scene.	
	TMB choice reaction time (Choice RT)	Cognitive test assessing psychomotor processing speed. Participants indicate the direction of the one arrow that is a different color from the rest of the arrows.	
	TMB matrix reasoning	Cognitive test assessing general cognitive ability and nonverbal reasoning. Participants solve a series of visual puzzles.	
	TMB paced serial addition task (PSAT)	Cognitive test assessing sustained attention and working memory. Participants add pairs of numbers that appear one after another and determine whether the sum is >10 or <10.	
	TMB flicker change detection (Flicker)	Cognitive test assessing visual working memory. Participants view a series of visual scenes with blue and yellow dots. One of the dots is changing color from blue to yellow. Participants are asked to indicate the dot that is changing color.	
	TMB adaptive delay discounting	Cognitive test assessing decision-making. Participants indicate whether they would prefer differencing amounts of hypothetical money now vs. in the future.	
	TMB visual paired associates memory – learn	Cognitive test assessing visual memory. Participants learn a set of picture pairs.	
	TMB multiple object tracking (MOT)	Cognitive test assessing sustained visual attention. Participants remember and track a set of target circles as they move around the screen among a larger set of identical distractor circles.	
	TMB visual paired associates memory – test	Cognitive test assessing episodic memory. Participants indicate which pictures go together based on the set they learned.	
	TMB letter-number switching	Cognitive test assessing cognitive flexibility and task switching. Participants indicate which response fits the instruction cue shown on screen.	
**Baseline (and EMA^c^) voice survey (approximately 5 min)**			
	Short sentence	Speech test assessing speech abnormalities. Participants repeat the following sentence: “The quick brown fox jumps over the lazy dog.”	
	Sustained phonation	Speech test assessing vocal instability. Participants take a deep breath in and then say the vowel “aaa” for 30 s, taking breaks as needed.	
	Diadochokinetic task	Speech test assessing articulation. Repeat “pah-tah-kah“ as many times as you can in 10 s.	
	Paragraph (amusement park)	Speech test assessing speech patterns and abnormalities. Participants are instructed to read the following paragraph about a day at the amusement park.	
	Feeling question	Question asking the participant to share how they are feeling and why for 1 min.	
	Semantic fluency	Speech test assessing verbal fluency. Participants are given 1 minute to come up with as many words as they can that fit the given category.	

^a^PROMIS: Patient-Reported Outcomes Measurement Information System.

^b^TMB: TestMyBrain.

^c^EMA: ecological momentary assessment.

#### Cognitive EMA, Speech EMA, and Blood-Based Biomarkers

##### Overview

Ultrabrief versions of the TMB full length cognitive tests were selected for the optimization pilot; these versions of the full-length tests demonstrate robust reliability and good sensitivity [37]. These specific cognitive tests were chosen based on their domain of assessment; that is, given processing speed and executive functioning are implicated in PKU, brief versions of cognitive tests measuring these domains were chosen for the EMA study portion. All tests were developed using a combination of JavaScript and HTML, delivered through web apps that were downloaded to the participants local device, ran in the browser, and then delivered data back to a central server. Analyses required participants to complete at least 4 assessments, 1 of which was the baseline assessment. A brief description of the tests used are below, with further detailed information and psychometric characteristics described in Germine et al [38] and Singh et al [28].

##### TMB Digit Symbol Matching

In digit symbol matching (DSM) [22,26,39], participants are presented with 6 symbols, each of which are paired with a single digit between 1 and 3 (ie, 2 symbols were paired with each digit). These pairings remain visible throughout the duration of the test. Individual probe symbols are sequentially presented above these pairings, to which patients respond by selecting the corresponding digit as quickly as possible. Each probe symbol remains visible until the patient makes a response. Scores are recorded as the total number of correct responses in 90 seconds. The primary test score of interest used for psychometric analyses is number of correctly completed matches (DSM.score).

##### TMB Gradual Onset Continuous Performance Test

In gradual onset continuous performance test (gradCPT) [40], the participant presses a key when a city image appears and does not press it when a mountain image appears. Images rapidly transition from one to the next, with mountains appearing only 10%-20% of the time. Scores are recorded as a measure of response bias where a larger value indicates greater response impulsivity, or tendency to press a key regardless of the picture type. The primary test score of interest and used for psychometric analyses is response bias (CPT.dprime).

##### TMB Multiple Object Tracking Test

In multiple object tracking (MOT) test [41], the participant remembers and tracks a set of target circles as they move around the screen, among a larger set of identical distractor circles. The primary test score of interest and used for psychometric analyses is percent correct (MOT.score).

Speech EMA includes the same speech tests used in the baseline voice survey (refer to Table 1 for a comprehensive list, tests’ relative duration, and response style, ie, repetition or free response).

To determine Phe and Tyr levels, participants were supplied with test kits from PerkinElmer that were mailed to their homes. Participants provided blood samples that were mailed directly to PerkinElmer after obtaining a fasting sample via finger prick on the morning of their scheduled EMA.

In addition to cognitive EMA, speech EMA, and blood biomarkers, we also collected passive measures, including metadata about browser, screen size, and operating system. This information was used in data analysis to ensure consistent data quality throughout participant responses.

#### Procedure

**Figure 1:**
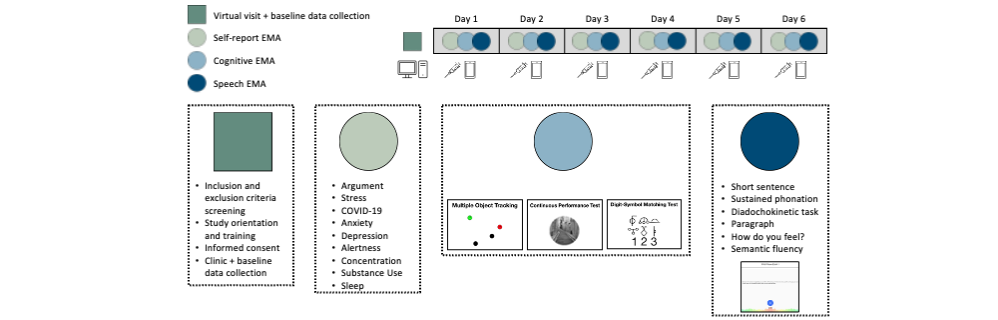
Study design. Overview of the study design for participants with phenylketonuria. EMA: ecological momentary assessment; PKU: phenylketonuria.

This study was compliant with ethical principles and approved by the Mass General Brigham (MGB) Institutional Review Board (IRB) and the NPKUA Ethics Committee. All participants completed an orientation and signed the informed consent form through the secure REDCap platform. In total, 23 participants were recruited through flyers distributed by the NPKUA. Participants were asked to complete 6 EMAs over 1 month, varying by day of the week (weekday vs weekend) and time of day (morning, afternoon, or evening). Ultrabrief versions of selected baseline tasks, based on areas of functioning typically impaired in individuals with PKU, were administered on a mobile device at varying times throughout the day to minimize participant burden. More specifically, EMAs were sent on predetermined days throughout the month (week 1: Wednesday; week 2: Tuesday and Friday; week 3: Monday and Sunday; and week 4: Thursday). All participants followed the same EMA schedule in Eastern Standard Time: EMA 1 at 10:13 AM, EMA 2 at 10:13 AM, EMA 3 at 7:45 PM, EMA 4 at 12:05 PM, EMA 5 at 1:46 PM, and EMA 6 at 5:57 PM. There was a minimum of 3 days and a maximum of 6 days between EMAs. All EMAs were delivered between 9 AM and 9 PM local time to reduce interference with participants’ daily schedules and sleep routines. All participants completed EMAs on their personal devices (smartphones). They were routinely sent push notifications that enabled them to access the assessments via a web link; they had 60 minutes to complete the assessment. Participants were compensated US $300 (per EMA and blood collection). The between-person reliability of each EMA task was evaluated, which is especially relevant given that each task was administered in real-life settings with a clinical population. Refer to Figure 1 for the overall study design.

The overall study design is the same as the optimization pilot discussed in this paper. Conducted over 1 month, the study includes a virtual prestudy visit conducted over Zoom (screening for inclusion and exclusion criteria, orientation, informed consent, and baseline assessment). From Days 1-6, participants complete repeated EMAs across three domains: (1) self-report EMAs that track variables such as argument occurrence, stress, COVID-19 impact, anxiety, depression, alertness, concentration, substance use, and sleep, (2) cognitive EMAs that include tasks like MOT, gradCPT, and DSM to measure cognitive function, and (3) speech EMAs include tasks such as short sentence repetition, sustained phonation, diadochokinetic exercises, paragraph reading, responses to “How do you feel?” prompts, and semantic fluency tasks.

### Ethical Considerations

This study adhered to ethical guidelines for human participants research as outlined by McLean Hospital, Harvard Medical School, and MGB. Before study initiation, a formal review of the research protocol was conducted by the MGB IRB. Based on the nature of the study, the IRB determined that no exemptions were applicable. Once the study documents were approved (IRB approval number 2024P001922), all research activities were conducted in full compliance with institutional policies and federal regulations to uphold ethical standards.

Before providing consent, participants received a detailed informed consent form that outlined key components of the study, such as the purpose, procedures, risks, benefits, confidentiality, and privacy rights. Participants were given adequate time to review the consent form, ask questions, and address concerns with study staff before providing consent. The consent process was conducted virtually through the secure online platform REDCap.

To safeguard participant privacy and confidentiality, each participant was assigned a unique participant ID (eg, PKU1) generated in REDCap during the virtual consent process. Identifiable information was securely stored in password-protected files, accessible only to authorized study staff, and all data were deidentified. All TMB data were automatically backed up nightly, with access restricted to authorized users. Data collected via SurveyLex, a Health Insurance Portability and Accountability Act (HIPAA)–compliant software administered by Sonde Health, has been approved for use in other MGB IRB protocols (eg, 2019P003458 and 2019P002752). Any data that our team retrieved from the SurveyLex website were encrypted in transit and stored securely in a designated Partners Dropbox folder.

Participants were compensated up to US $300 for their participation in the study. Compensation included US $10 for each brief daily assessment (6 assessments × US $10 = US $60) and US $40 for each blood collection (6 collections × US $40 = US $240). Payments were issued via checks mailed to the participants’ homes.

### Statistical Analyses

EMA and baseline tasks were parsed using Python 3.11 via Spyder IDE 5.4.3. Analysis was done using R 4.3.1 [42] in RStudio 2023.06.1+524 [43], with the packages tidyverse [44], plyr [45], and psych [46]. Plots were made with ggplot, part of tidyverse, as well as patchwork [47]. The between-person reliability of each EMA task was evaluated, which is especially relevant given that each task was administered in real-life settings with a clinical population. The between-person reliability, which represents the variance that is due to differences between individuals versus within individuals, was assessed using 2 different approaches: the mlr (multilevel reliability) function from the psych package in R (for gradCPT, MOT, and DSM) and a regression-based approach (for semantic fluency, due to the missing trial structure), modeled after Mascarenhas Fonseca et al [37]. The mlr function computes reliability as well as generalizability, using unconditional multilevel mixed models to predict performance for each EMA, with nested within-person random effects (between-person reliability: mlr ‘RkRn’; within-person reliability mlr ‘Rcn’ [24]). For gradCPT EMAs, response bias (dprime) was calculated for odd and even trials, and performance was predicted for the other half of the EMA. For DSM, scores were also calculated based on odd and even trials [27]. For MOT, scores were calculated on a trial-by-trial level [21] based on previous works [21,48]. EMA number and trial number (in case of MOT) or for both halves of the task (gradCPT and DSM) were coded from –2.5 to 2.5.

For the regression-based approach, using the lmer function in r, the calculated test scores were used and entered into an unconditional multilevel mixed model (equation 1) to predict scores on each EMA. Fitting the model allowed partitioning of variance, which could then be entered into equation 2 [37]:

semanticFluency<–lmer(score~1 + (1 + EMA_num|participant),data = df) **(1)**

EMA_num was a vector coded from –2.5 to 2.5 for EMAs 1 to 6.

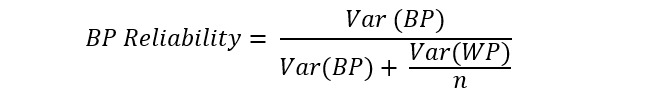

Where Var (BP) is the total variance in scores between participants, Var (WP) is the variance in scores within participants (ie, variance between EMA sessions and residual variance), and n is the total number of measurements (in this case, the mean number of EMAs completed across all participants) [48]. Between-person reliability was reported for each EMA task, together with average completion rate and average EMA performance.

To assess variability in task performance (in percent) over the course of the EMAs, the coefficient of variability (CV) was calculated (equation 3) for each of the 3 tasks.

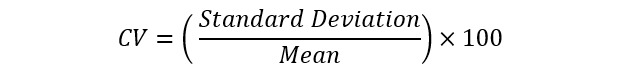



To make sure that we understand if performance was impacted by time of day, we used *t* tests to compute whether there was a significant difference in performance between morning EMAs (EMAs 1 and 2), evening (EMA 3 and 6), and morning and midday EMAs (EMA 4 and 5).

Finally, for baseline tasks, average performance was reported. Notably, although we collected self-reported mood and psychological data, given the small sample size, further data will need to be collected to sufficiently power additional analyses regarding the relationship among EMA and mood or self-report.

## Results

### Participants

In total, 21 adults with PKU were enrolled in this study between December 2022 and March 2023 (Table 2 illustrates descriptive statistics); however, only 20 participants were included in the EMA analyses due to 1 participant’s participation in a very similar study that was not disclosed before enrollment. Following the exclusion of that participant, 2 participants had missing baseline data, so 18 total participants were included in analyses. All 20 participants were diagnosed with PKU at or around the time of birth, and diagnosis details were self-reported. Among the participants, 19 had classic PKU (Phe >1200 mmol/L), 1 had hyperphenylalanemia (Phe 120-600 mmol/L), and 3 were unsure of their specific diagnosis type.

**Table 2:** Description of study sample (N=20).

Characteristic				Values
Age (years), mean (SD)				37.24 (11.33)
**Sex, n (%)**				
		Female		12 (60)
		Male		8 (40)
**Race or ethnicity, n (%)**				
	American Indian or Alaskan Native		0 (0)	
	Asian		0 (0)	
	African or Black		0 (0)	
	Native Hawaiian or Pacific Islander		0 (0)	
	European or White		20 (100)	
	Hispanic or Latino		0 (0)	
**Education, n (%)**				
	Primary school (less than 7 years)		0 (0)	
	Middle or junior high school (7-10 years)		0 (0)	
	Secondary school (high school diploma or GED^a^)		2 (10)	
	Some college or University		3 (15)	
	Technical training or associate degree		2 (10)	
	Bachelor’s degree		5 (25)	
	Master’s degree		5 (25)	
	Graduate or professional degree (eg, PhD, MD, and JD)		3 (15)	

^a^GED: General Educational Development.

### Baseline Results

Participants performed several baseline tasks that will be used for further analysis in subsequent publications as the study continues. Descriptive results are reported in Table 3 and presented alongside normative data from the TMB database for each test, respectively. Data in the table are reported as mean (SD) across the entire participant sample (for this study data as well as the normative sample). This is true for all score and accuracy data but also for the mean and median reaction times data.

**Table 3:** Baseline descriptive results.

Cognitive test and task measure			PKU^a^ pilot sample				TMB^b^ normative data		
			Participants, n		Result, mean (SD)		Participants, n		Result, mean (SD)
**TMB MOT^c^**			18				1931		
	Accuracy			78.55 (9.32)				79.86 (10.22)	
	Score			56.56 (6.71)				57.5 (7.36)	
**TMB letter or number switching^d^**			18				3421		
	Accuracy			98.55 (1.96)				96.46 (0.66)	
	Mean RTc^e^			1169.44 (270.50)				1252.61 (364.43)	
	Median RTc			1060.74 (249.63)				1134.07 (349.32)	
**TMB delay discounting^f^**			18				24,081		
	ln(k)^g^			–6.22 (1.68)				–4.73 (2.18)	
**TMB choice RTc**			18				34,641		
	Score			15.32 (21.22)				11.22 (3.51)	
	Accuracy			87.41 (27.21)				93.44 (13.48)	
	Mean RTc			1009.82 (252.23)				969.07 (317.23)	
	Median RTc			964.28 (222.79)				912.02 (290.51)	
**TMB flicker^h^**			18				13,363		
	Score			11.61 (3.72)				10.79 (6.11)	
	Accuracy			96.58 (9.19)				94.31 (0.93)	
	RTc Mean			6927.79 (1961.00)				7271.13 (2347.85)	
	RTc Median			5859.93 (1973.97)				6028.51 (2314.93)	
	flipscMean^i^			9.42 (2.85)				9.90 (3.37)	
	flipscMedian			7.91 (2.78)				8.13 (3.32)	
**TMB paced serial addition task^j^**			18				3835		
	Score			46.44 (7.72)				44.59 (11.03)	
	Accuracy			77.41 (12.86)				74.32 (11.03)	
	Mean RTc			881.82 (146.04)				825.09 (181.44)	
	Median RTc			852.79 (161.77)				797.57 (196.34)	
**TMB SRT^k^**			18				60,874		
	Mean RTc			341.40 (111.7)				317.67 (72.05)	
	Median RTc			323.08 (94.72)				304.35 (67.70)	
**TMB visual paired associates memory task^l^**			18				9758		
	Score			15.5 (5.73)				15.86 (4.57)	
	Accuracy			63.23 (23.41)				66.1 (19.04)	
	Mean RTc			3941.48 (819.75)				3850.91 (840.13)	
	Median RTc			3639.84 (843.8)				3561.11 (972.49)	
**TMB matrix reasoning^m^**			18				25,829		
	Score			26.06 (4.77)				27.26 (5.91)	
	Accuracy			74.44 (13.63)				82.80 (0.9)	
	Mean RTc			7275.12 (2209.59)				10,098.3 (5905.12)	
	Median RTc			5039.36 (1366.61)				6056.32 (2829.13)	
**TMB vocabulary^n^**			18				36,230		
	Score			26.78 (1.59)				23.22 (5.52)	
	Accuracy			86.7 (5.96)				77.4 (18.41)	
	Mean RTc			3544.74 (778.43)				7279.86 (3607.23)	
	Median RTc			3164.19 (856.14)				5718.68 (2520.94)	
**TMB gradCPT^o^**			18				4669		2.70 (0.76)
	dprime^p^			2.64 (0.74)					

^a^PKU: Phenylketonuria.

^b^TMB: TestMyBrain website [49].

^c^MOT: multiple object tracking (visuospatial attention and visual working memory). A higher score or accuracy indicates better performance.

^d^Letter or number switching: switching between 2 tasks, testing response selection or inhibition. Higher accuracy indicates better performance.

^e^RTc: reaction time (processing speed and response selection or inhibition). A higher score indicates higher processing speed. A higher accuracy indicates better performance. The measures are shown in ms.

^f^Delay discounting: adaptive delay discounting, choosing between smaller immediate and larger delayed rewards (temporal discounting and impulsivity). Delay discounting is measured using the natural logarithm of the discounting factor k (ln(k)). ln(k) is negative for small values, indicating more future-oriented individuals, and positive for larger k values, indicating more impulsive, immediate-reward focused individuals.

^g^ln(k): natural logarithm of the discounting factor k.

^h^Flicker: flicker change detection (visual search, change detection, and visual working memory). A higher accuracy indicates better performance. The same is true for the score.

^i^number of image flips for correct responses to “test” trials (ms).

^j^Paced serial addition: adding pairs of numbers, sustained attention, working memory. A higher score (number of correct trials) and accuracy (proportion of correct trials) indicate better performance.

^k^SRT: simple reaction time (psychomotor response speed). The mean and median correct response times are indicated, and shorter response times indicate faster processing speed.

^l^Visual paired associates memory: visual memory, episodic memory (remembering pictures). A higher score (number of correct trials) and accuracy (proportion of correct trials) indicate better performance.

^m^Matrix reasoning: fluid cognitive ability and nonverbal reasoning. A higher score (number of correct trials) and accuracy (proportion of correct trials) indicate better performance.

^n^Vocabulary: identifying synonyms (crystallized cognitive ability and verbal reasoning). A higher score (number of correct trials) and accuracy (proportion of correct trials) indicate better performance.

^o^gradCPT: gradual onset continuous performance test. Performance is measured using dprime. Dprime can be interpreted as the discrimination sensitivity in the task, with higher values indicating a better ability to perform the task.

^p^dprime: response bias.

### Biomarkers

The averages for Phe, Tyr, and the Phe:Tyr ratio were recorded daily for each scheduled EMA. These blood-based biomarkers were measured in micromoles per liter (mmol/L), as illustrated in Table 4.

**Table 4:** Phenylalanine, tyrosine, and phenylalanine:tyrosine ratio averages by day of ecological momentary assessment administration.

Day				Values, mean (SD)
**Day 1 (n=17)**				
	Phe^a^			425.18 (429.43)
	Tyr^b^			43.92 (19.13)
	Phe:Tyr ratio			10.66 (11.13)
**Day 2 (n=18)**				
	Phe			480.61 (504.59)
	Tyr			42.41 (11.44)
	Phe:Tyr ratio			11.76 (12.98)
**Day 3 (n=17)**				
	Phe			482.98 (448.82)
	Tyr			42.41 (14.61)
	Phe:Tyr ratio			11.52 (10.53)
**Day 4 (n=17)**				
		Phe		478.17 (456.04)
		Tyr		39.47 (13.19)
		Phe:Tyr ratio		13.02 (10.02)
**Day 5 (n=17)**				
			Phe	515.36 (432.93)
			Tyr	38.36 (11.83)
			Phe:Tyr ratio	12.61 (10.20)
**Day 6 (n=14)**				
		Phe		524.23 (360.14)
		Tyr		43.91 (12.33)
		Phe:Tyr ratio		11.85 (6.85)

^a^Phe: phenylalanine.

^b^Tyr: tyrosine.

### EMA Results

Participants were prompted to complete 4 tasks as part of each of the 6 EMAs (“measurement time points”) in the study. On average, each task was completed between 4.78 times out of the 6 measurement time points. Between-person reliability (ie, the consistency of the differences in scores between individuals [27]) was slightly lower or comparable to previous EMA studies [27,40] specifically for the TMB MOT and TMB DSM, and comparable with or exceeding a representative sample from TestMyBrain [40,49]. However, while reliability was slightly lower for the TMB gradCPT (0.72) and semantic fluency (0.70), they still fall within a good range (reliability between 0.4 and 0.59 is considered fair, 0.60 and 0.74 good, and reliability above 0.75 excellent). Results are listed in Table 5.

**Table 5:** Initial reliability data for ecological momentary assessments, based on the data collected from the phenylketonuria pilot sample.

Outcome	PKU^a^ pilot sample			
	Participants, n	Mean (SD)	Between-person reliability of the EMA^b^	Within-person reliability of the EMA
Brief TMB^c^ gradCPT^d^—dprime^e^	20	2.66 (0.75)	0.72	0
Brief TMB DSM^f^—score (#correct)	20	23.18 (4.07)	0.93	0.87
Brief TMB MOT^g^—accuracy	20	71.37 (22.13)	0.88	0
Brief semantic fluency—score	20	20.97 (7.91)	0.70	—^h^

^a^PKU: phenylketonuria.

^b^EMA: ecological momentary assessment.

^c^TMB: TestMyBrain website [49].

^d^gradCPT: gradual onset continuous performance test.

^e^dprime: response bias.

^f^DSM: digit symbol matching.

^g^MOT: multiple object tracking.

^h^Not available (due to missing trial structure, this measure cannot computed for this task, because there is no variance within each individual ecological momentary assessment).

**Figure 2:**
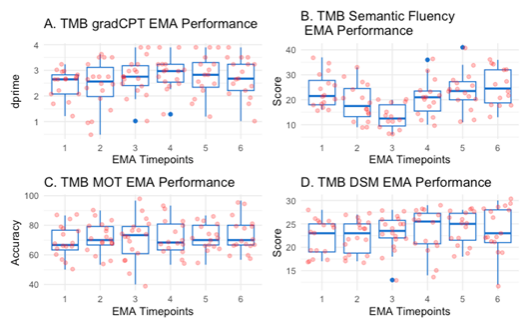
Ecological momentary assessment performance across cognitive tasks. dprime: response bias; DSM: digit symbol matching; EMA: ecological momentary assessment; gradCPT: gradual onset continuous performance test; MOT: multiple object tracking; TMB: TestMyBrain website.

Figure 2 provides an overview of participant performance in all 4 tasks that were administered as part of an EMA, over the course of the 6 EMAs. Boxplots depict the data distribution, with the median shown as a horizontal line, the lower and upper quartile values as the edges of the box, while the whiskers represent the minimum and maximum data values. The blue points represent outliers (defined as the standard to be above or below the upper or lower quartile value minus 1.5 times the IQR) and the red points show individual participant values.

Overview of participants’ performance on individual cognitive tasks administered as part of each EMA conducted over 1 month. The x-axes represent EMA time points from day 1 to day 6, and the y-axes represent participant performance for each task. Panel A shows performance on the gradCPT (n=20), with dprime values shown on the y-axis. The gradCPT assesses sustained attention, and participants are required to distinguish between target and nontarget stimuli. Dprime can be interpreted as the discrimination sensitivity in the task, with higher values indicating a better ability to perform the task. Panel B shows performance on the semantic fluency task (n=20), where the y-axis represents participants’ ability to produce words within a specific category (eg, animals) during a set time frame. A higher score indicates better semantic fluency. Panel C shows performance on the MOT (n=20), with the y-axis reflecting participants’ accuracy. The MOT assesses speeded visual attention, and participants are asked to track a set of target circles. A higher number indicates better accuracy, meaning how well participants tracked objects. Finally, panel D shows performance on the Digit Symbol Matching (DSM; n=20), with the y-axis representing the number of correctly completed matches. The DSM measures processing speed, where participants match symbols to corresponding digits as quickly as possible. The EMAs were sent on predetermined days throughout the month (week 1, EMA 1: Wednesday; week 2, EMA 2 and 3: Tuesday and Friday; week 3, EMA 4 and 5: Monday and Sunday; and week 4, EMA 6: Thursday). All participants followed the same EMA schedule in Eastern Standard Time: EMA 1 at 10:13 AM (gradCPT, MOT, and DSM, 17 out of 20 participants completed, verbal fluency: 16 out of 20 participants completed EMA), EMA 2 at 10:13 AM (gradCPT, MOT, and DSM, 18 out of 20 participants completed, verbal fluency: 16 out of 20 participants completed EMA), EMA 3 at 7:45 PM (gradCPT, MOT, and DSM, 18 out of 20 participants completed, verbal fluency: 14 out of 20 participants completed EMA), EMA 4 at 12:05 PM (gradCPT, MOT, and DSM, 16 out of 20 participants completed, verbal fluency: 15 out of 20 participants completed EMA), EMA 5 at 1:46 PM (gradCPT, MOT, and DSM, 16 out of 20 participants completed, verbal fluency: 16 out of 20 participants completed EMA), and EMA 6 at 5:57 PM (gradCPT, MOT, and DSM, 17 out of 20 participants completed, verbal fluency: 14 out of 20 participants completed EMA). There was a minimum of 3 days and a maximum of 6 days between EMAs. A total of 12 participants resided in the Eastern Time Zone, 4 in the Central Time Zone, 1 in the Mountain Time Zone, and 3 in the Pacific Time Zone.

Quantitative assessment of variability indicates a coefficient of variability of 28% for the gradCPT, 37% for semantic fluency, 15.8% for the MOT, and 17.6% for the DSM. Hence, performance is more stable in those tasks measuring processing speed, visual short-term memory, and visuospatial attention (DSM and MOT), while performance on the gradCPT (measuring sustained attention, response inhibition, and cognitive control) as well as semantic fluency (also measuring executive control) is much more variable over time. The semantic fluency prompt (ie, “name everything you can think of in this category”) was changed for each EMA, and therefore greater variability was expected for this speech-based task. This task also represents domains (eg, verbal fluency and aspects of executive functioning) known to be difficult for patients with PKU [50]. Performance in the TMB gradCPT is measured using dprime, which indicates the discrimination sensitivity in the task. Higher values reflect a better ability to perform the task. Performance in the TMB MOT is measured using accuracy, which assesses the ability to correctly track objects. Median performance across all EMAs is at 70%, indicating participants’ general ability to do well in the task. Furthermore, TMB DSM performance is measured using the DSM score, which indicates the number of correctly identified matches in the task. Higher values indicate a higher processing speed and better visual short-term memory. Median performance appears stable across all EMA measurements, with little fluctuation.

Finally, we assessed whether performance was significantly different between the morning (EMA 1 and 2) and evening (EMA 3 and 6) and morning and midday (EMA 4 and 5) EMAs. Results showed that for the DSM, there was no significant difference in performance between the morning and evening (*t*_13.72_*=*1.46, *P*=.17) or morning and midday EMAs (*t*_13.90_=–0.98, *P*=.34; average performance morning: mean 23, SD 3.76; midday: mean 24.94, SD 4.11; evening: mean 25.56, SD 3.26). For semantic fluency, we again did not see a difference in mean performance between morning (mean 19.2, SD 4.73) and evening (mean 19.3, SD 4.75) EMAs (*t*_8_=–0.033, *P*=.97) and morning and midday (mean 20.4, SD 5.18) EMAs (*t*_7.93_=–0.38, *P*=.71). A similar pattern emerges for the MOT, with no significant difference between morning (percent correct: mean 71.46, SD 19.35) and evening (mean 76.04, SD 19.54) EMAs (*t*_94_=–1.16, *P*=.25) or morning and midday (mean 74.79, SD 19.46) EMAs (*t*_94_=–0.84, *P*=.40). Finally, the same is true for the comparison between morning (dprime: mean 2.83, SD 0.32) and evening (mean 3.07, SD 0.51) EMAs in the gradCPT (*t*_11.68_=–1.13, *P*=.29), and between the morning and midday (mean 3.17, SD 0.40) performance (*t*_13.25_=–1.86, *P*=.09). Hence, performance does not decrease when EMAs are administered later in the day compared with earlier (all reported *t* tests are 2-tailed).

### Attrition

In total, 21 participants were recruited through the NPKUA. Furthermore, 1 participant was removed because of a conflict of interest (they were a part of another study with a laboratory we are collaborating with at the University of Missouri-Columbia). All participants completed the study in its entirety. Regarding EMAs, completion rates were around 70% (between 4.6 and 5 measurements out of 6); 1 participant only completed 1 EMA and 2 blood samples.

## Discussion

### Principal Findings

Individuals with PKU face a large health care burden given that they endure multiple hospital visits from childhood, and must consistently monitor diet and blood levels, without the advent of continuous monitoring digital tools [51,52]. These individuals are more likely to experience chronic conditions of organ systems, further increasing their health care burden [52,53]. In addition, given that PKU is inherently a rare disease, there is often a dearth of specialty providers who can routinely monitor patients with the disease; thus, the time burden on caregivers and patients alike is further exacerbated [51]. Studies on this population need to focus on scalable, accessible ways of remotely monitoring individuals with PKU, so they can achieve a better quality of life with less health care burden. Thus, EMA studies in this population fill a unique gap, wherein providers and researchers alike can continuously monitor patients at scale, enabling greater access to individuals with PKU both nationally and internationally. Smartphones and the widespread availability of personal digital devices further facilitate larger-scale studies that can readily incorporate novel or experimental measures as they are developed.

The goal of this pilot study was to (1) demonstrate that EMA is a valid and reliable methodology for evaluating fluctuations in cognitive status in individuals diagnosed with PKU and (2) optimize a test battery that can be iterated on in the larger protocol based on these results. Given that this is the first study examining cognitive and speech EMA in individuals diagnosed with PKU, there were limited previous studies on which to develop appropriate frequency or timepoint decisions. Therefore, we turned to other clinical studies that use EMA in clinical and community samples [24,37]; based on the results demonstrating robust reliability (between-person for all tests and within-person for processing speed), it appears that this frequency can be deemed appropriate for this population.

Results suggest that EMAs were completed adequately well in this clinical sample, with completion rates above 70% (4.78 measurements out of 6). Furthermore, performance in both EMA measures and baseline tests appears in line with expectations based on expansive normative data, both for community and clinical samples, which demonstrate participants can complete these types of tasks with relatively low attrition or participant burden. Reliability is also close to what has been recorded in other samples, though slightly lower for the gradCPT and semantic fluency (0.7 and above), with the latter, however, representing a domain that is known to be specifically difficult for patients with PKU [53]. The TMB DSM and TMB MOT tests demonstrate the strongest between-person reliability in this sample, consistent with previous studies and the TMB representative sample [37].

Notably, there appeared to be small “dips” in semantic fluency on EMA 3; however, this qualitative decline cannot only be accounted for by time of assessment (ie, evening), given that EMA 6 was also in the evening, and time of day did not significantly impact performance. Furthermore, we did not see a significant difference between EMA 3 (mean 15.6, SD 3.85) and 6 (mean 23, SD 7.12; *t*_6.16_=–2.05, *P*=.09). Similarly, TMB MOT appears to be somewhat more variable during EMA 3 as well, so we compared variability in performance on EMA 3 versus EMA 6, given that they are both administered in the evening, and there was no significant difference in performance (*t*_93.98_=–0.28, *P*=.78) between EMA 3 (mean 75.41, SD 21.92) and EMA 6 (mean 76.67, SD 21.57). In addition, performance on the MOT during EMA 3 was in no way significantly different (Fligner-Killeen test of homogeneity of variances: *P*=.93). Therefore, it appears that there may be natural peaks and troughs in performance that, although not statistically significant, strengthen the argument for this methodology, which enables one to assess fluctuations in performance over one full month.

### Limitations

Future work might examine whether higher-frequency EMAs could potentially offer greater utility in capturing nuanced changes in everyday cognition. Previous work by Mascarenhas Fonseca et al [37] has demonstrated that 3 EMAs per day for 10 days was particularly effective in capturing changes in glucose levels in individuals diagnosed with type 1 diabetes; this frequency should be examined within the PKU population, with finger prick tests completed at the time of EMA, rather than first thing in the morning, as done in this study, but no conclusions were drawn based on blood-based biomarkers given the limited range of Phe or Tyr. Ongoing analyses are determining whether any specific changes in blood levels within individuals may offer some interesting insights in relation to fluctuations in cognitive status. In addition, given the small sample size and relatively (racially or ethnically) homogeneous population, it is difficult to determine the generalizability of the results. All participants were recruited through the NPKUA, and therefore special efforts aimed at encouraging diversity were limited. Future work might attempt to recruit individuals with PKU from the broader community to better capture diversity. As this study continues, obtaining a larger sample size, though difficult in a rare disease population, will be critical in determining how cognition, other speech characteristics (besides semantic fluency), and blood-based biomarkers (Phe, Tyr) interact. Collecting this information will facilitate a data-driven approach in streamlining and refining the battery used in this study.

### Conclusion

The EMA pilot study described in this manuscript demonstrated the psychometric reliability and feasibility of EMA studies in individuals with PKU. By leveraging digital tools, EMA offers the ability to remotely capture everyday cognitive functioning, outside of a single time point of assessment. The digital nature of EMA batteries facilitates entirely remote test administration, enabling more rapid and scalable patient monitoring while improving equity and accessibility, particularly in clinical trials interested in outcome-based research. Future projects may focus on validating a singular battery that could be rapidly administered at scale and in clinical trials to determine the progression of disease, with or without pharmacological intervention.

## Abbreviations

BPR: between-person reliability
dprime: response bias
DSM: digit symbol matching
EMA: ecological momentary assessment
gradCPT: gradual onset continuous performance test
HIPAA: Health Insurance Portability and Accountability Act
IRB: Institutional Review Board
MGB: Mass General Brigham
mlr: multilevel reliability
MOT: multiple object tracking
NPKUA: National PKU Alliance
Phe: phenylalanine
PHEFREE: Phenylalanine Families and Researchers Exploring Evidence
PKU: phenylketonuria
REDCap: Research Electronic Data Capture
TMB: TestMyBrain website

## Data Availability

The datasets generated during or analyzed during this study are not publicly available due to participant confidentiality and privacy concerns but are available from the corresponding author on reasonable request.
